# The fatty liver index exhibits a dual association with chronic obstructive pulmonary disease: a machine learning-based analysis of two independent cohorts

**DOI:** 10.3389/fnut.2026.1752300

**Published:** 2026-05-05

**Authors:** Yiben Huang, Zihan Ye, Xinran Li, Ruizi Xu, Yiting Yu, Rong Yu, Fan Xu, Xiuxiu Zhao, Xianjing Chen, Chunyan Liu, Beibei Yu, Yage Xu, Xiaodiao Zhang

**Affiliations:** 1 Department of Respiratory and Critical Medicine, The Third Affiliated Hospital of Wenzhou Medical University, Wenzhou, China; 2 The Second School of Medicine, Wenzhou Medical University, Wenzhou, Zhejiang, China; 3 Renji College, Wenzhou Medical University, Wenzhou, Zhejiang, China; 4 The First School of Medicine, School of Information and Engineering, Wenzhou Medical University, Wenzhou, China

**Keywords:** BODE, COPD, FLI, machine learning, MASLD

## Abstract

**Background:**

Chronic obstructive pulmonary disease (COPD) and non-alcoholic fatty liver disease share common metabolic and inflammatory pathways. This study aims to elucidate the dual association of the fatty liver index (FLI) with COPD.

**Methods:**

A dual-cohort study was conducted using data from the National Health and Nutrition Examination Survey (NHANES) 2017–2020 (*n* = 15,560) and a clinical cohort from the Wenzhou Third Affiliated Hospital, China (*n* = 296). Feature selection was performed using three machine learning algorithms. Multivariate logistic regression, restricted cubic splines were used to examine associations. The receiver operating characteristic curves, confusion matrix evaluated model accuracy. A nomogram was developed to visualize the contribution of key predictive factors for COPD.

**Results:**

After adjustment for machine learning-selected covariates, FLI was positively associated with COPD prevalence in the NHANES cohort (OR = 1.013, 95% CI: 1.005–1.021, *p* = 0.002) but inversely associated with COPD severity in the Wenzhou cohort (OR = 0.567, 95% CI: 0.336–0.955, *p* = 0.033). A non-linear threshold relationship was identified in the Wenzhou cohort (*p*-non-linear = 0.002), with an inflection point at FLI = 1.045. Machine learning-optimized models demonstrated good predictive accuracy, with the NHANES model achieving an AUC of 0.819.

**Conclusion:**

FLI exhibits a dual association with COPD, serving as a risk factor for its prevalence in the general population yet being inversely associated with disease severity among diagnosed patients. These findings support FLI as a clinically relevant, non-invasive biomarker for stratifying COPD risk and assessing severity.

## Introduction

Chronic obstructive pulmonary disease (COPD), a leading cause of global morbidity and mortality, is characterized by progressive airflow limitation and persistent respiratory symptoms, placing a significant burden on healthcare systems (1, 2). Notably, COPD is frequently accompanied by multiple metabolic comorbidities, including non-alcoholic fatty liver disease (NAFLD)—a condition recently redefined as metabolic dysfunction–associated steatotic liver disease (MASLD) to better reflect its cardiometabolic underpinnings (3, 4).

MASLD ranks among the most prevalent metabolic disorders worldwide, characterized by excessive lipid accumulation in hepatocytes that is not attributable to alcohol consumption or other well-defined causes of liver injury (4, 5). The pathogenesis and progression of MASLD are intrinsically associated with inflammatory processes, wherein inflammatory mediators contribute critically to dysregulated lipid metabolism and hepatocellular injury (6). Similarly, inflammation, oxidative stress, and metabolic dysregulation are recognized as underlying mechanisms contributing to the development and progression of COPD (7, 8). Emerging evidence suggests that MASLD and COPD share partially overlapping pathological mechanisms related to disordered lipid metabolism, indicating potential crosstalk between the liver and lungs through a “liver-lung axis” at the metabolic level (9, 10). However, evidence in this area remains limited, warranting further investigation.

We employed the fatty liver index (FLI) as a metric to assess the progression and severity of MASLD (11). This non-invasive and readily calculable indicator has been widely adopted in clinical and epidemiological studies (12). It has also been validated as being associated with a variety of metabolic disorders, including cardiovascular diseases, hypertension and insulin resistance (13–15). However, its association with the progression of COPD remains unclear. Given the shared metabolic and inflammatory pathways implicated in the pathogenesis of both COPD and MASLD, we hypothesized that FLI could serve as a novel biomarker for assessing the prevalence and severity of COPD.

This dual-cohort study aimed to comprehensively evaluate the association between the FLI and both the risk and severity of COPD. A cohort from the National Health and Nutrition Examination Survey (NHANES) was used to assess the association between FLI and COPD prevalence, whereas a clinical cohort from the Wenzhou Third Affiliated Hospital served to examine the link between FLI and disease severity among patients with established COPD. Using machine learning-based variable selection to develop predictive models, we sought to determine whether FLI, a non-invasive marker of metabolic dysfunction, could serve as a practical biomarker for early risk stratification and severity assessment in COPD, thereby providing novel insights into the liver-lung axis and facilitating personalized clinical management strategies.

## Materials and methods

### Study population

This retrospective study comprised two distinct cohorts: 15,560 participants from the NHANES database (2017–2020) and 296 COPD patients admitted to the Third Affiliated Hospital of Wenzhou Medical University from February 1, 2018 to July 5, 2022.

The first cohort constituted individuals from the 2017-2020 NHANES database. Eligibility for inclusion was determined by applying the following criteria: (1) age > 40 years; (2) those with data on COPD; (3) those without asthma. The criteria for participant exclusion in our study included: (1) those with hepatic insufficiency, severe renal dysfunction, cancer or pregnancy; (2) presence of chronic hepatitis such as viral hepatitis or autoimmune hepatitis; (3) missing data necessary for FLI calculations; (4) those without relevant covariates.

The population of the second study cohort was obtained from the Third Affiliated Hospital of Wenzhou Medical University between February 1, 2018 and July 5, 2022. Inclusion criteria were as follows: (1) age > 40 years; (2) being diagnosed with COPD by a pulmonologist; (3) forced expiratory volume in 1 second (FEV_1_)/forced vital capacity (FVC) < 70%; (4) those with the body mass index, airflow obstruction, dyspnea, exercise capacity (BODE) index data within 7 days of admission; (5) those without asthma; (6) those with data necessary for FLI calculations. We excluded patients with severe hepatic insufficiency, severe renal dysfunction, chronic hepatitis, cancer, and pregnancy or incomplete data on covariates.

### Data collection and definitions

The NHANES is a biennial US survey assessing health and nutritional status through interviews, examinations, and laboratory tests. The study protocol received approval from the NCHS Research Ethics Review Board. Additional details regarding the dataset utilized in this investigation are accessible through the official NHANES website.^1^ We extracted various data of the subjects in the NHANES database, including demographic characteristics, anthropometric information, clinical data, as well as clinical examination data. Baseline and demographic data for the hospital cohort were collected from electronic medical records.

### Assessment of FLI

FLI is a fatty liver prediction index calculated from basic clinical and laboratory indicators, including triglyceride (TG) levels, body mass index (BMI), γ-glutamyl transferase (GGT), and waist circumference (WC), using the following formula (11): FLI = (e^0.953 *ln(*TG*) + 0.139 * *BMI* + 0.718 * *ln*(*GGT*) + 0.053 * *WC* – 15.745^)/(1 + e^0.953 * *ln*(*TG*) + 0.139 * *BMI* + 0.718 * *ln*(*GGT*) + 0.053 * *WC* –15.745^) * 100. Where ln is the natural logarithm, e is the base of the natural logarithm, TG is in mmol/L, GGT is in U/L, WC is in cm, and scores range from 0 to 100.

### Outcomes

In the NHANES cohort, COPD was defined by a self-reported physician diagnosis, specifically an affirmative response to the question: “Has a doctor or other health professional ever told you that you had COPD?” In the clinical cohort from the Third Affiliated Hospital of Wenzhou Medical University, disease severity was the primary outcome, categorized based on the BODE index. Participants with a BODE index score ≥ 5 were classified into the more severe COPD category (16).

### Covariates

Our covariates in the NHANES cohort included age, sex, marital status (married or living with a partner, Never married, Widowed/divorced/separated), educational level (below high school, high school, and above high school), ratio of family income to poverty (PIR) (< 1.5, 1.5–4.0, ≥ 4.0), alcohol status (yes or no), smoking status (yes or no), prior medical history (cardiovascular disease (CVD), hypertension, heart disease, and diabetes), and clinical indicators including triglycerides, total cholesterol (TC), alanine aminotransferase (ALT), aspartate aminotransferase (AST), alkaline phosphatase (ALP), platelets, monocytes, lymphocytes, eosinophil granulocyte and other clinical indicators.

Smoking status was classified based on whether participants had a lifetime consumption of at least 100 cigarettes, while alcohol use was defined as any reported consumption of alcoholic beverages. Hypertension diagnosis was established through three separate blood pressure measurements, with an average systolic pressure ≥ 140 mmHg or diastolic pressure ≥ 90 mmHg indicating hypertensive status. Diabetes mellitus and heart disease were ascertained through self-reported medical history. CVD was determined by a medical condition questionnaire, mainly by the affirmative answers to any of the following five questions: “Ever told had congestive heart failure?,” “Ever told you had coronary heart disease?,” “Ever told you had angina/angina pectoris?,” “Ever told you had heart attack?,” and “Ever told you had a stroke?.”

Patient age, sex, smoking, alcohol status, marital status (married, widowed/divorced/separated), educational level (illiterate, primary school, junior high school and high school), household per capita annual income (HPCI) (<10,000, 10,000–30,000, 30,000–50,000, 50,000–100,000, >100,000), history of hypertension, diabetes and heart disease and clinical indicators including triglycerides, TC, ALT, AST, ALP, platelets, monocytes, lymphocytes, eosinophil granulocyte, total cholesterol, and other clinical indicators were extracted as covariates in the cohort of the Third Affiliated Hospital of Wenzhou Medical University.

### Statistical analysis

Normality was assessed using the Shapiro-Wilk test; normally distributed data are expressed as mean ± standard deviation, and skewed data as median interquartile range (IQR). Categorical variables were analyzed using chi-square tests, and continuous variables were analyzed using *t*-tests or Mann-Whitney U tests. We used multiple imputation to handle missing covariate data in the Wenzhou cohort.

Spearman correlation heat maps were used to exclude highly correlated covariates. The variance inflation factor (VIF) was used to identify and eliminate variables with high collinearity. Machine learning methods (including LASSO, Boruta, and XGBoost) were used to conduct variable screening, incorporate the selected data into multivariate logistic regression analysis, and explore the relationship between FLI and COPD prevalence/severity after adjusting for confounding factors. LASSO was chosen for sparse linear selection via L1 regularization, Boruta for capturing non-linear relationships through random forest-based shadow feature comparison, and XGBoost for modeling complex feature interactions via gradient boosting—together ensuring comprehensive and robust variable screening. Detailed parameter settings for the three machine learning algorithms are provided in Supplementary methods.

Restricted cubic spline (RCS) curves and threshold analysis were used to explore the non-linear relationship between FLI and COPD prevalence. The receiver operating characteristic (ROC) curves, net reclassification improvement (NRI), and integrated discrimination improvement (IDI) index were used to evaluate model accuracy. Furthermore, the model’s performance was evaluated using a confusion matrix, from which accuracy, sensitivity, specificity, positive predictive value (PPV), negative predictive value (NPV) and F1-score were calculated. Then, a nomogram was constructed using the variables selected by machine learning to display the relative importance of these factors for predicting COPD prevalence. Finally, the diagnostic performance of the machine learning model and the full-variable model were evaluated using the ROC curve, calibration curves, decision curve analysis (DCA) and precision-recall (PR) curves. Statistical significance was set at *p* < 0.05 (two-sided). Data were analyzed using SPSS 24.0 and RStudio 4.4.1.

## Results

### Multiple imputation and correlation analysis of covariates

In Supplementary Figure 1, to address missing covariate data in the Wenzhou cohort, multiple imputation was employed. Supplementary Figure 1A shows that variables such as the length of hospitalization, neutrophil count, monocyte count, lymphocyte count, platelet count and eosinophil count had missing values, as indicated by the prominent red portions of the bars in the chart. The heatmap legend shows the correspondence between the color and the correlation coefficient, where blue and red denote negative and positive correlations, respectively, with color intensity proportional to the strength of the association. In Supplementary Figure 1C, a significant correlation was observed between male sex and smoking in the Wenzhou cohort (*r* = 0.868).

### Covariate selection by machine learning

**FIGURE 1:**
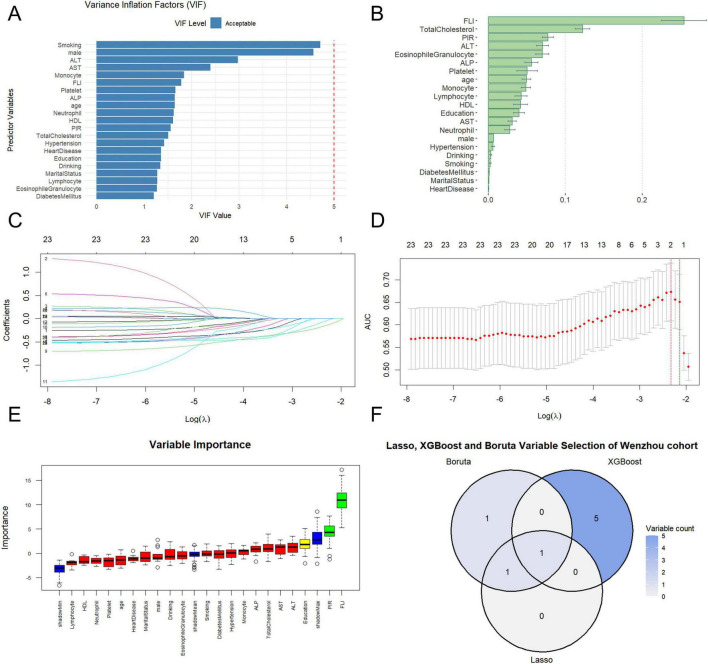
FLI feature selection of COPD in the Wenzhou cohort. **(A)** VIF detection was performed for all variables. **(B)** Variable importance plot for the XGBoost model. **(C)** LASSO regression path plot. **(D)** LASSO 10-fold cross-validation plot. **(E)** The figure shows the results of feature selection after computerized processing by the Boruta algorithm. **(F)** Three algorithmic Venn diagram screening variables.

Before establishing the machine learning models, we used the VIF to evaluate the degree of collinearity between variables. By identifying and excluding variables with a VIF > 5 value, we could simplify the model structure. As shown in Figure 1A and Supplementary Figure 2A, there was no multicollinearity among our variables in both cohorts.

Figure 1 shows the machine learning covariate selection results of the Wenzhou cohort. We used the XGBoost algorithm to screen out the following variables: FLI, total cholesterol, HPCI, ALT, eosinophil granulocyte, and ALP. Figure 1B shows the importance of these variables. Through computation, we obtained the variables selected by LASSO, as follows: HPCI, FLI (Figures 1C,D). We used the Boruta algorithm to screen out the following variables: HPCI, FLI, education (Figure 1E). Finally, we used a Venn diagram to show the overlapping relationship of covariates through the three machine learning algorithms described above in Figure 1F.

The machine learning covariate selection results of the NHANES cohort are shown in Supplementary Figure 2. The variables selected by the XGBoost algorithm are as follows: smoking, age, platelet, CVD, FLI, ALT (Supplementary Figure 2B). We used the LASSO algorithm to screen out the following variables in Supplementary Figures 2C,D: age, marital status, hypertension, diabetes mellitus, PIR, CVD, smoking, neutrophils, monocytes, ALP, FLI. We obtained the variables selected by Boruta, as follows: CVD, smoking, FLI, age, ALT, heart disease, lymphocytes, neutrophils, total cholesterol, AST, monocytes, ALP (Supplementary Figure 2E). In Supplementary Figure 2F, the Venn diagram shows the overlapping relationship of covariates through the three machine learning algorithms described above.

**TABLE 1:** Characteristics of the COPD grouped according to BODE.

	Total (*n* = 143)	BODE < 5 (*n* = 92)	BODE ≥ 5 (*n* = 51)	*p*-value
Demographics				
Age (years)	70.64 ± 7.91	70.71 ± 8.05	70.53 ± 7.74	0.899
Male (*n%*)	120 (83.9)	78 (84.8)	42 (82.4)	0.705
Marital status (*n%*)				0.711
Married	128 (89.5)	83 (90.2)	45 (88.2)	
Widowed, divorced, or separated	15 (10.5)	9 (9.8)	6 (11.8)	
Education (*n%*)				0.193
Illiterate	52 (36.3)	32 (34.8)	20 (39.2)	
Primary school	67 (46.9)	40 (43.5)	27 (52.9)	
Junior high school	20 (14.0)	17 (18.5)	3 (5.9)	
High school	4 (2.7)	3 (3.3)	1 (2.0)	
HPCI (*n%*)				**0.028**
<10,000	42 (29.4)	20 (21.7)	22 (43.1)	
10,000–30,000	51 (35.7)	32 (34.8)	19 (37.3)	
30,000–50,000	25 (17.5)	19 (20.7)	6 (11.8)	
50,000–100,000	20 (14.0)	17 (18.5)	3 (5.9)	
>100,000	5 (3.5)	4 (4.3)	1 (2.0)	
Body measurement indicators				
Height (cm)	162.50 ± 7.15	163.26 ± 7.47	161.12 ± 6.38	0.086
Weight (kg)	56.64 ± 9.59	59.75 ± 9.10	51.02 ± 7.78	**<0.001**
BMI (kg/m^2^)	21.43 ± 3.32	22.42 ± 3.17	19.65 ± 2.83	**<0.001**
Waist circumference (cm)	88.29 ± 9.02	89.88 ± 8.45	85.42 ± 9.39	**0.004**
Medical history				
Hypertension (*n%*)	62 (43.4)	44 (47.8)	18 (35.3)	0.147
Diabetes mellitus (*n%*)	27 (18.9)	21 (22.8)	6 (11.8)	0.105
Heart disease (*n%*)	19 (13.3)	12 (13.0)	7 (13.7)	0.908
Smoking (*n%*)	114 (79.7)	75 (81.5)	39 (76.5)	0.472
Drinking (*n%*)	65 (45.5)	44 (47.8)	21 (41.2)	0.444
Blood indicators				
Platelet (10^9^/L)	211.00 (174.00–279.00)	206.00 (172.50–270.00)	214.00 (175.00–284.00)	0.733
Neutrophil (10^9^/L)	3.90 (2.90–5.90)	3.95 (2.80–5.98)	3.90 (2.90–5.50)	0.726
Monocyte (10^9^/L)	0.50 (0.40–0.80)	0.60 (0.40–0.72)	0.50 (0.30–0.80)	0.525
Lymphocyte (10^9^/L)	1.40 (1.10–1.80)	1.40 (1.10–1.88)	1.40 (1.00–1.80)	0.642
Eosinophil granulocyte (10^9^/L)	0.14 (0.07–0.21)	0.15 (0.08–0.24)	0.13 (0.06–0.17)	0.253
Total cholesterol (mmol/L)	4.21 ± 0.89	4.26 ± 0.91	4.11 ± 0.87	0.349
HDL-C (mmol/L)	1.05 (0.92–1.28)	1.03 (0.86–1.27)	1.10 (0.96–1.31)	0.140
GGT (U/L)	26.00 (19.00–44.00)	26.50 (20.25–43.50)	23.00 (16.00–45.00)	0.127
ALT (U/L)	15.00 (10.00–21.00)	15.00 (11.00–24.00)	13.00 (10.00–19.00)	**0.042**
AST (U/L)	19.00 (16.00–24.00)	19.00 (16.00–25.00)	18.00 (13.00–22.00)	0.073
ALP (U/L)	75.00 (61.00–89.00)	75.00 (62.50–90.75)	74.00 (57.00–86.00)	0.409
Triglycerides (mmol/L)	0.94 (0.70–1.40)	1.02 (0.73–1.50)	0.82 (0.66–1.14)	**0.011**
Other related indicators				
FLI	0.32 (0.14–0.84)	0.45 (0.18–1.08)	0.18 (0.11–0.37)	**<0.001**

COPD, chronic obstructive pulmonary disease; HDL-C, high-density lipoprotein cholesterol; ALT, alanine aminotransferase; ALP, alkaline phosphatase; AST, aspartate aminotransferase; GGT, gamma-glutamyl transferase; BMI, body mass index; FLI, the fatty liver index; HPCI, household per capita annual income; BODE, body mass index, airflow obstruction, dyspnea, exercise capacity index. The baseline characteristics of the study participants from the NHANES cohort are summarized in Supplementary Table 1 stratified according to the presence or absence of COPD. Compared with patients without COPD, patients with COPD were older and had higher waist circumference, neutrophil count, monocyte count, ALP. Furthermore, patients with COPD had lower GGT, ALT and AST levels. Bold values indicate statistical significance (*p* < 0.05).

### Baseline characteristics of participants

Table 1 presents the baseline characteristics of participants in the Wenzhou cohort, stratified by COPD severity using a BODE score cutoff of 5. Patients with more severe COPD (BODE ≥ 5) exhibited lower anthropometric and metabolic parameters, including weight, BMI, waist circumference, triglycerides, and ALT, compared to those in less severe group. Most significantly, FLI values were substantially lower in the group with BODE scores ≥ 5 [0.18 (0.11–0.37) vs. 0.45 (0.18–1.08)], suggesting a potential association between MASLD and COPD prevalence (*p* < 0.001).

### Association between FLI and the prevalence and severity of COPD

We developed three logistic regression models to examine the association between FLI and both the prevalence and severity of COPD across both cohorts. The Wenzhou cohort models were adjusted to: Model 1: No adjustments were made; Model 2: Adjusted for HPCI (overlapping variables filtered by three machine learning methods); Model 3: Adjusted for HPCI, education, eosinophil granulocyte, ALP (all variables filtered by three machine learning methods).

The NHANES cohort models were adjusted to: Model 1: No adjustments were made; Model 2: Adjusted for CVD, smoking, age (overlapping variables filtered by three machine learning methods); Model 3: Adjusted for CVD, smoking, age, ALT, heart disease, lymphocyte, neutrophil, total cholesterol, AST, monocyte, ALP, platelet, marital status, hypertension, diabetes mellitus, PIR (all variables identified by three machine learning methods).

As shown in Table 2, we discovered that in the unadjusted Model 1, FLI correlated well with the prevalence and severity of COPD in both cohorts (Wenzhou: *p* = 0.015; NHANES: *p* = 0.001). In the Wenzhou cohort, FLI was independently associated with COPD severity and was negatively associated with the risk of COPD after adjusting for covariates (Model 2: OR = 0.559, 95% CI: 0.332–0.941, *p* = 0.029; Model 3: OR = 0.567, 95% CI: 0.336–0.955, *p* = 0.033). However, in the NHANES cohort, after adjusting for covariates, FLI was significantly and positively associated with the prevalence of COPD (Model 2: OR = 1.014, 95% CI: 1.007–1.021, *p* < 0.001; Model 3: OR = 1.013, 95% CI: 1.005–1.021, *p* = 0.002).

**TABLE 2:** Adjusted odds ratio (95% confidence interval) of FLI for COPD and BODE ≥ 5.

Variables	Model 1		Model 2		Model 3	
	OR (95% CI)	*p*-value	OR (95% CI)	*p*-value	OR (95% CI)	*p*-value
Wenzhou 2018.2.1–2022.7.5						
BODE ≥ 5	0.511 (0.296–0.879)	**0.015**	0.559 (0.332–0.941)	**0.029**	0.567 (0.336–0.955)	**0.033**
NHANES 2017–2020						
COPD	1.011 (1.004–1.017)	**0.001**	1.014 (1.007–1.021)	**<0.001**	1.013 (1.005–1.021)	**0.002**

COPD, chronic obstructive pulmonary disease; PIR, poverty-to-income ratio; HPCI, household per capita annual income; FLI, fatty liver index; BODE, body mass index, airflow obstruction, dyspnea, exercise capacity index. Bold values indicate statistical significance (*p* < 0.05).

### Non-linear relationship and threshold analysis results

**FIGURE 2:**
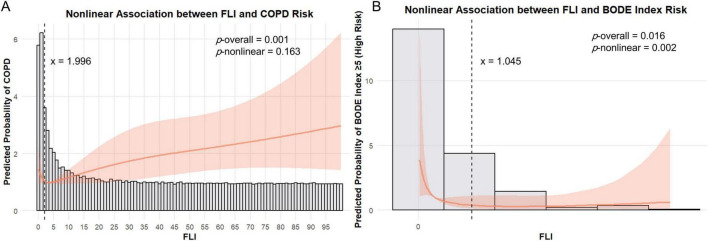
The non-linear association between FLI and COPD status and severity. **(A)** The restricted cubic spline (RCS) curve shows the association between FLI and COPD in the NHANES cohort. We did not conduct variable adjustments. **(B)** The RCS curve shows the association between FLI and BODE in the Wenzhou cohort. We did not conduct variable adjustments.

We constructed RCS curves without adjusting for covariates in Figure 2 and found that the probability of COPD increased with increasing FLI (*p* for overall = 0.001). There was a linear relationship between FLI and COPD prevalence (*p* for non-linear = 0.163) in the NHANES cohort (Figure 2A). However, in the Wenzhou cohort, the probability of COPD severity increasing decreased with higher FLI (*p* for overall = 0.016); there was a non-linear relationship between FLI and COPD severity (*p* for non-linear = 0.002) (Figure 2B). Therefore, we conducted a threshold analysis to examine the relationship between FLI and COPD severity in the Wenzhou cohort. Supplementary Table 2 demonstrates that a threshold effect exists between the continuous variable of FLI and the discrete variable of COPD (*p* for likelihood test = 0.007). When FLI values exceeded 1.045, no association was observed between FLI and COPD. However, when FLI values were ≤ 1.045, FLI showed a negative correlation with COPD (OR = 0.062; 95% CI = 0.005–0.842, *p* = 0.037).

### Predictive ability of different models for the prevalence and severity of COPD

The ROC analysis, NRI and IDI index were used to evaluate model accuracy in Table 3. ROC analysis showed that all models performed well in predicting the prevalence and severity of COPD (Wenzhou cohort: Model 1: *p* < 0.001; Model 2: *p* < 0.001; Model 3: *p* < 0.001; NHANES cohort: Model 2: *p* < 0.001; Model 3: *p* < 0.001), except Model 1 in the NHANES cohort (*p* = 0.218). In the NHANES cohort, in terms of predicting COPD prevalence, Model 3 had a 92.6% improvement in NRI (*p* < 0.001) and a 12.7% improvement in IDI (*p* < 0.001) compared to the Model 1, with better reclassification ability. The results of the Wenzhou cohort study showed that compared with Model 1, both Model 2 and Model 3 had better reclassification ability in predicting COPD severity. Model 2 NRI increased by 45.6% (*p* = 0.003) and IDI increased by 5.2% (*p* = 0.007), while for Model 3 NRI increased by 35.5% (*p* = 0.034) and IDI increased by 6.9% (*p* = 0.002), indicating that both models had good predictive performance. In Supplementary Table 3, we compared the performance of Model 2 and Model 3. Model 3 demonstrated superior diagnostic performance with an accuracy rate of 92.7%, sensitivity of 9.7%, specificity of 99.2%, PPV of 50.0%, NPV of 93.3%, and an F1-score of 16.2% in the NHANES cohort. In the Wenzhou cohort, Model 2 performed better with an accuracy rate of 64.3%, sensitivity of 26.7%, specificity of 85.2%, PPV of 50.0%, NPV of 67.6%, and an F1-score of 34.8%. Based on these findings, Model 3 from the NHANES cohort and Model 2 from the Wenzhou cohort were selected as the main models for subsequent construction of nomogram models.

**TABLE 3:** Reclassification and discrimination statistics of the models for predicting COPD and BODE ≥ 5.

	ROC-AUC		NRI		IDI	
Variables	Estimate (95% CI)	*p*-value	Estimate (95% CI)	*p*-value	Estimate (95% CI)	*p*-value
Wenzhou 2018.2.1–2022.7.5						
BODE ≥ 5						
Model 1	0.292 (0.204–0.381)	**<0.001**	Reference		Reference	
Model 2	0.713 (0.627–0.799)	**<0.001**	0.456 (0.159–0.753)	**0.003**	0.052 (0.014–0.089)	**0.007**
Model 3	0.746 (0.664–0.828)	**<0.001**	0.355 (0.254–0.685)	**0.034**	0.069 (0.026–0.113)	**0.002**
NHANES 2017–2020						
COPD						
Model 1	0.526 (0.482–0.570)	0.218	Reference		Reference	
Model 2	0.792 (0.762–0.822)	**<0.001**	0.930 (0.810–1.049)	**<0.001**	0.093 (0.076–0.109)	**<0.001**
Model 3	0.819 (0.791–0.848)	**<0.001**	0.926 (0.803–1.050)	**<0.001**	0.127 (0.106–0.148)	**<0.001**

COPD, chronic obstructive pulmonary disease; AUC, area under the curve; NRI, net reclassification index; IDI, integrated discrimination improvement; PIR, poverty-to-income ratio; HPCI, household per capita annual income; FLI, fatty liver index; BODE, body mass index, airflow obstruction, dyspnea, exercise capacity index. Bold values indicate statistical significance (*p* < 0.05).

### Evaluation of the nomogram model

**FIGURE 3:**
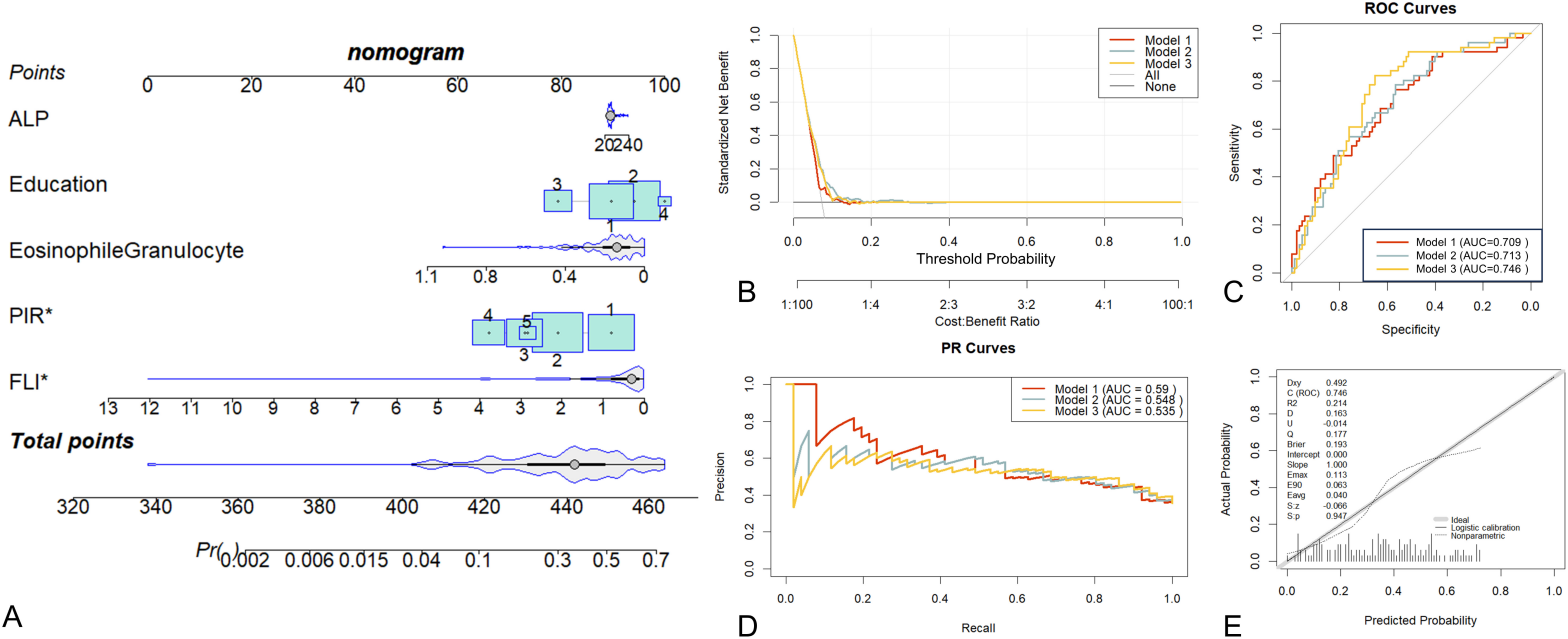
Nomogram for the diagnosis of BODE. **(A**) Nomogram for the diagnosis of BODE. **(B)** Decision Curve. **(C)** ROC Curve **(D)** PR curve for diagnosis of BODE. **(E)** Calibration curve for prediction accuracy. The Wenzhou cohort models were adjusted to: Model 1: No adjustments were made; Model 2: Adjusted for HPCI (overlapping variables filtered by three machine learning methods); Model 3: Adjusted for HPCI, education, eosinophil granulocyte, ALP (all variables filtered by three machine learning methods).

We aimed to construct nomogram models using FLI and these biomarkers to predict the risk of COPD prevalence and the severity of COPD. In the Wenzhou cohort, a nomogram was constructed for Model 3 to visualize the prediction of BODE diagnosis (Figure 3A), incorporating five variables: ALP, education, eosinophil granulocyte, PIR, and FLI. The total points corresponded to predicted probabilities from 0.002 to 0.7. Model validation demonstrated good discriminative ability (ROC curve, Figure 3C; Model 3: AUC = 0.746), favorable calibration (calibration curve, Figure 3E; Hosmer–Lemeshow test *p* = 0.578; standardized residual *p* = 0.594), and clinical utility (decision curve analysis, Figure 3B). Precision-recall analysis (Figure 3D) showed moderate performance for Model 2 (AP = 0.548) and Model 3 (AP = 0.535). In the NHANES cohort, the nomogram (Supplementary Figure 3A) incorporated 14 variables with total points ranging from 1,000 to 1,160. A 40-point increase in FLI corresponded to a 28% increase in COPD risk. Model 3 demonstrated good calibration (H-L test *p* = 0.647; standardized residual *p* = 0.258), favorable discriminative ability (AUC = 0.819), and acceptable clinical utility compared to Model 2 (Supplementary Figures 3B–E).

### Sensitivity analyses

The robustness of our findings was corroborated through multiple sensitivity analyses. First, analyses employing commonly recognized confounding factors produced results consistent with our primary findings, mitigating concerns about potential overadjustment due to machine learning-selected variables (Supplementary Table 4). Second, the stability of our results was further confirmed using overlap weighting—a method that achieves excellent covariate balance by focusing on the population with overlapping propensity scores (Supplementary Table 5 and Supplementary Figure 4). Third, sensitivity analyses applying validated FLI cutoffs demonstrated a graded positive association between FLI categories and COPD prevalence in the NHANES cohort, providing additional evidence for the robustness of our findings (Supplementary Table 6). Fourth, to further enhance the credibility of our results, we performed K-fold cross-validation, which yielded estimates largely consistent with our primary analyses and confirmed the stability of model performance (Supplementary Figure 5). Finally, bootstrap validation with 1,000 resamples was conducted to assess the stability of the identified threshold. Bootstrap resampling (1,000 iterations) yielded a median threshold of 0.381 (95% CI: 0.042–1.833), with the original threshold of 1.045 falling within this interval. The significant log-likelihood ratio test (LLR = 7.417, *p* = 0.025) further supports the presence of a threshold effect (Supplementary Table 7).

## Discussion

This study is the first to systematically investigate the dual association between FLI and both the prevalence and severity of COPD. It employs a dual-cohort design to enhance the comprehensiveness and generalizability of the findings. Our findings indicate that FLI is significantly associated with both the prevalence and severity of COPD, although the direction of this association varies across populations.

In the NHANES cohort, elevated FLI was positively associated with increased COPD prevalence, which we attribute to multiple interconnected biological pathways common to both COPD and MASLD (17, 18). Systemic inflammation and oxidative stress represent core shared mechanisms. In MASLD, hepatic lipid accumulation triggers the release of pro-inflammatory cytokines (TNF-α, IL-6) and reactive oxygen species, which enter the systemic circulation and may promote pulmonary inflammation and tissue remodeling (19). Conversely, in COPD, chronic airway inflammation generates systemic inflammatory mediators that can exacerbate hepatic steatosis and fibrosis, creating a bidirectional crosstalk (20). Lipid metabolism disturbances play a central role in both conditions. Alterations in phospholipids, cholesterol, and fatty acids are associated with changes in pulmonary cellular function and surfactant composition in COPD (21, 22). Cholesterol metabolites may impair liver X receptor-dependent cholesterol efflux in COPD lung tissue, leading to impaired macrophage apoptosis and dysregulated suppression of lipopolysaccharide-induced and bacteria-induced proinflammatory cytokine expression, thereby potentially exacerbating COPD severity (23–25). Meanwhile, the accumulation of free cholesterol in the liver increases the risk of developing MASH and fibrosis in patients with MASLD (26, 27).

The liver-lung axis involves complex molecular crosstalk beyond simple inflammatory spillover. Hepatocyte-derived extracellular vesicles carrying lipids, microRNAs, and inflammatory mediators may travel via the circulation to the lung, modulating alveolar macrophage function and epithelial cell homeostasis (28, 29). Emerging evidence also implicates the fibroblast growth factor 21 (FGF21)—adiponectin axis as a potential endocrine link, with FGF21 produced by the liver in response to metabolic stress exerting pleiotropic effects on pulmonary tissues (30, 31). Furthermore, the gut-liver-lung axis adds another layer of complexity (32). MASLD-associated gut dysbiosis and increased intestinal permeability may facilitate the translocation of bacterial products (e.g., lipopolysaccharides) into the circulation, contributing to systemic inflammation that affects both hepatic and pulmonary compartments (33). These overlapping pathways may help explain the observed association between FLI and COPD prevalence.

The observed negative correlation between FLI and COPD severity in the Wenzhou cohort may reflect the “obesity paradox.” Specifically, obesity and visceral adipose tissue accumulation may exert a potentially protective effect in certain chronic diseases, including MASLD (34–36). Obesity is associated with reduced lung volumes and may confer a mechanical advantage in COPD patients through a potential “chest wall strapping” effect, which can help protect small airways and reduce the risk of progression to emphysema and the risk of mortality (37–40). Furthermore, obese patients may possess greater nutritional and metabolic reserves, which could help mitigate the catabolic stress during acute exacerbations of COPD, consistent with previous reports (41–43).

The divergent incremental value of Model 3 over Model 2 between cohorts reflects fundamental differences in population heterogeneity and statistical power. In NHANES, the general population exhibited wide variability in metabolic and inflammatory profiles, enabling the additional clinical and laboratory covariates in Model 3 to substantially enhance discrimination of COPD prevalence. In contrast, the Wenzhou cohort comprised hospitalized patients with established COPD, diminishing the marginal contribution of these covariates. Furthermore, the limited sample size (*n* = 143) and event rate (*n* = 51) in Wenzhou constrained stable estimation of additional model parameters, rendering the more parsimonious Model 2 preferable for this context. Notably, the inclusion of BMI in both Model 2 and Model 3 may introduce mathematical coupling that could further confound comparisons in this severity-specific analysis (16). Moreover, the relationship between FLI and COPD severity exhibited significant non-linearity with a threshold effect (*p* = 0.002), which linear models may not capture adequately. These findings underscore that optimal model specification is highly context-dependent, varying with target population characteristics and available data resources.

A key strength and innovation of this study lies in the integration of three complementary machine learning algorithms (LASSO, Boruta, and XGBoost) for high-dimensional variable selection. This data-driven approach minimized subjective bias and mitigated overfitting, enabling the identification of robust predictors that might have been overlooked by conventional methods. Furthermore, our evaluation framework extended beyond traditional performance metrics by incorporating reclassification indices (NRI, IDI) and F1-score, alongside a comprehensive suite of four analytic curves—ROC curves, calibration curves, decision curve analysis, and PR curves. This multi-faceted assessment allowed us to thoroughly evaluate not only predictive accuracy and discriminative ability, but also model calibration and clinical utility—an aspect often neglected in similar studies.

While this study provides valuable insights through a dual-cohort design and machine learning approaches, several important limitations should be acknowledged. First, the retrospective design of both cohorts limits the ability to establish causality. Second, although advanced machine learning methods were employed to screen and adjust for confounding factors, residual bias from unmeasured or unknown factors cannot be excluded. Moreover, despite assessing collinearity using the VIF, the machine learning-based selection process itself may carry an inherent risk of overadjustment. Third, the diagnosis of COPD in the NHANES cohort was based on self-report without spirometric confirmation. This approach may introduce misclassification bias, potentially underestimating true effect sizes if non-diseased individuals were incorrectly classified as having COPD, or vice versa. Our findings therefore require validation in cohorts with objectively confirmed COPD diagnoses. Fourth, there are substantial differences in the demographic characteristics between the two cohorts. Fifth, the Wenzhou cohort comprised a relatively small sample size, and the threshold analysis was exploratory with inherent variability in estimation. Furthermore, the cohort exclusively comprised patients diagnosed with COPD by clinicians, lacking a non-COPD control group that underwent the same clinical measurements, thereby precluding a case-control analysis. Consequently, our findings warrant caution and require validation through case-control analyses in larger, multicenter studies involving spirometry-confirmed COPD patients.

## Conclusion

In conclusion, this dual-cohort study reveals a dual role of FLI in COPD, indicating that elevated FLI is a risk factor for disease prevalence in the general population but is associated with less severe clinical progression among diagnosed patients. Utilizing machine learning for robust variable selection, we demonstrated that FLI, as a simple, non-invasive biomarker, holds potential for improving risk stratification and personalized management in COPD. Further prospective studies are warranted to validate these findings and elucidate the underlying mechanisms.

## Data Availability

Publicly available datasets were analyzed in this study. Comprehensive details about the NHANES database are publicly available at https://wwwn.cdc.gov/nchs/nhanes/Default.aspx. The data from the Third Affiliated Hospital of Wenzhou Medical University are not publicly available to ensure patient confidentiality. However, the data are available from the corresponding author upon reasonable request.

## Footnotes

1
https://www.cdc.gov/nchs/nhanes/index.htm
