# Supplementary material for: The fatty liver index exhibits a dual association with chronic obstructive pulmonary disease: a machine learning-based analysis of two independent cohorts
Source: Front Nutr. 2026 May 5;13:1752300. doi: 10.3389/fnut.2026.1752300 (PMC13185564; doi:10.3389/fnut.2026.1752300)
Supplement: Supplementary file 1 [file Table_1.docx]

**SUPPLEMENTARY MATERIALS**

**The Fatty Liver Index Exhibits a Dual Association with COPD: A Machine Learning-Based Analysis of Two Independent Cohorts**

**Short Title:** FLI and COPD: A Dual-Cohort Study

**Supplementary Methods**

LASSO regression was implemented using the “glmnet” package in R. Given the binary nature of the outcome variable, the model was fitted with family = "binomial" and alpha = 1 to enforce pure LASSO penalization. Optimal regularization was determined via 10-fold cross-validation, and variables with non-zero coefficients at lambda.min—the λ value minimizing cross-validation error—were retained as selected features.

The Boruta algorithm was employed as an all-relevant feature selection method using the “Boruta” package. The algorithm was configured with *p*-Value = 0.01 and maxRuns = 500, running until either the maximum number of iterations was reached or variable importance stabilized. Features consistently confirmed as statistically significant across iterations were deemed important.

The XGBoost algorithm was applied to complement the linear and random forest-based approaches by capturing complex non-linear relationships and feature interactions. Key hyperparameters were set as follows: eta = 0.01 (learning rate), max_depth = 3 (maximum tree depth), gamma = 0.3 (minimum loss reduction required for node split), lambda = 2.0 (L2 regularization weight), and alpha = 0.3 (L1 regularization weight). To address class imbalance in the outcome variable, oversampling was performed using the ovun.sample function from the “ROSE” package prior to model training. Feature importance was quantified through 5-fold cross-validation, calculated as the mean Gain (average improvement in accuracy contributed by each feature) across folds, with standard errors computed to assess stability.

**Supplementary Tables**

**Supplementary Table 1.** Characteristics of the participants according to COPD

|  | Total (*n* = 2846) | without COPD (*n* = 2639) | with COPD (*n* = 207) | *p*-value |
| --- | --- | --- | --- | --- |
| **Demographic information** |  |  |  |  |
| Age (years) | 59.00 (50.00-67.00) | 58.00 (49.00-66.00) | 65.00 (58.00-73.00) | **< 0.001** |
| Male (*n* %) | 1472 (51.7) | 1354 (51.3) | 118 (57.0) | 0.114 |
| Marital status (*n* %) |  |  |  | **< 0.001** |
| Married or living with a partner | 1776 (62.4) | 1680 (63.7) | 96 (46.4) |  |
| Never married | 820 (28.8) | 726 (27.5) | 94 (45.4) |  |
| Widowed, divorced, or separated | 250 (8.8) | 233 (8.8) | 17 (8.2) |  |
| Education (*n* %) |  |  |  | **< 0.001** |
| < High school | 530 (18.6) | 484 (18.3) | 46 (22.2) |  |
| High school | 695 (24.4) | 620 (23.5) | 75 (36.2) |  |
| > High school | 1621 (57.0) | 1535 (58.2) | 86 (41.5) |  |
| Poverty-to-income ratio (*n* %) |  |  |  | **< 0.001** |
| ≤ 1.5 | 845 (29.7) | 759 (28.8) | 86 (41.5) |  |
| 1.5-4.0 | 1125 (39.5) | 1035 (39.2) | 90 (43.5) |  |
| > 4.0 | 876 (30.8) | 845 (32.0) | 31 (15.0) |  |
| **Body Measurement Indicators** |  |  |  |  |
| Height (cm) | 166.20 (159.20-173.80) | 166.10 (159.20-173.90) | 167.00 (160.30-172.80) | 0.579 |
| Weight (kg) | 80.80 (69.10-95.70) | 80.60 (69.00-95.50) | 83.10 (69.40-99.40) | 0.261 |
| BMI (kg/m^2^) | 28.96 (25.44-33.52) | 28.93 (25.47-33.41) | 29.55 (25.23-35.25) | 0.259 |
| Waist circumference (cm) | 101.20 (92.10-111.20) | 101.00 (92.00-111.50) | 105.70 (94.00-119.30) | **< 0.001** |
| **Medical history** |  |  |  |  |
| Hypertension (*n* %) | 638 (22.4) | 597 (22.6) | 41 (19.8) | 0.350 |
| Diabetes mellitus (*n* %) | 560 (19.7) | 489 (18.5) | 71 (34.3) | **< 0.001** |
| Cardiovascular disease (*n* %) | 307 (10.8) | 239 (9.1) | 68 (32.9) | **< 0.001** |
| Heart disease (*n* %) | 160 (5.6) | 125 (4.7) | 35 (16.9) | **< 0.001** |
| Smoking (*n* %) | 1239 (43.5) | 1075 (40.7) | 164 (79.2) | **< 0.001** |
| Drinking (*n* %) | 2588 (90.9) | 2390 (90.6) | 198 (95.7) | **0.014** |
| **Blood indicators** |  |  |  |  |
| Platelet (10^9^/L) | 233.00 (198.00-276.00) | 234.00 (199.00-276.00) | 227.00 (189.00-275.00) | 0.131 |
| Neutrophil (10^9^/L) | 3.90 (3.00-4.90) | 3.90 (2.90-4.90) | 4.60 (3.40-5.50) | **< 0.001** |
| Monocyte (10^9^/L) | 0.50 (0.40-0.70) | 0.50 (0.40-0.70) | 0.60 (0.50-0.70) | **< 0.001** |
| Lymphocyte (10^9^/L) | 2.10 (1.60-2.60) | 2.10 (1.60-2.60) | 2.00 (1.50-2.60) | 0.097 |
| Eosinophil Granulocyte (10^9^/L) | 0.20 (0.10-0.20) | 0.20 (0.10-0.20) | 0.20 (0.10-0.30) | 0.069 |
| Total cholesterol (mmol/L) | 120.00 (87.00-172.00) | 121.00 (87.00-172.00) | 117.00 (90.00-172.50) | 0.846 |
| HDL-C (mmol/L) | 1.32 (1.09-1.60) | 1.32 (1.11-1.60) | 1.29 (1.06-1.50) | 0.051 |
| GGT (IU/L) | 22.00 (15.00-34.00) | 22.00 (15.00-34.00) | 19.00 (14.00-30.00) | **0.005** |
| ALT (U/L) | 18.00 (13.00-25.00) | 18.00 (14.00-26.00) | 15.00 (12.00-21.00) | **< 0.001** |
| AST (IU/L) | 20.00 (16.00-24.00) | 20.00 (16.00-24.00) | 18.00 (15.00-23.00) | **0.004** |
| ALP (U/L) | 75.00 (63.00-91.00) | 75.00 (62.00-91.00) | 82.00 (66.00-97.00) | **< 0.001** |
| Triglycerides (mmol/L) | 1.36 (0.98-1.94) | 1.37 (0.98-1.94) | 1.32 (1.02-1.95) | 0.846 |
| **Other related indicators** |  |  |  |  |
| FLI | 2.57 (0.75-9.54) | 2.56 (0.76-9.27) | 2.87 (0.71-19.68) | 0.218 |

Abbreviations: COPD, chronic obstructive pulmonary disease; HDL-C, high-density lipoprotein cholesterol; ALT, alanine aminotransferase; ALP, alkaline phosphatase; AST, aspartate aminotransferase; GGT, gamma-glutamyl transferase; BMI, body mass index; FLI, fatty liver index.

**Supplementary Table 2**. Threshold effect analysis of FLI on COPD severity using a two-piecewise linear regression model

| Outcome | Effect | |
| --- | --- | --- |
|  | OR (95% CI) | *p*-value |
| Fitting by standard linear model | 0.511 (0.272-0.821) | **0.016** |
| Fitting by two-piecewise linear model |  | |
| Inflection point | 1.045 |  |
| < 1.045 | 0.062 (0.005-0.842) | **0.037** |
| > 1.045 | 1.500 (0.801-2.808) | 0.205 |
| Log-likelihood ratio | 9.807 | **0.007** |

Abbreviations: COPD, chronic obstructive pulmonary disease; FLI, fatty liver index.

**Supplementary Table 3.** Comparative performance of model prediction

|  | Model 2 | Model 3 |
| --- | --- | --- |
| **Wenzhou 2018.2.1-2022.7.5** | |  |
| BODE ≥ 5 |  |  |
| Accuracy | 64.3% | 57.1% |
| Sensitivity | 26.7% | 20.0% |
| Specificity | 85.2% | 77.8% |
| PPV | 50.0% | 33.3% |
| NPV | 67.6% | 63.6% |
| F1 Score | 34.8% | 25.0% |
| **NHANES 2017-2020** | |  |
| COPD |  |  |
| Accuracy | 92.6% | 92.7% |
| Sensitivity | 3.2% | 9.7% |
| Specificity | 99.6% | 99.2% |
| PPV | 40.0% | 50.0% |
| NPV | 92.9% | 93.3% |
| F1 Score | 6.0% | 16.2% |

The NHANES cohort model is adjustd to: Model 1: No adjustments were made; Model 2: Adjusted for CVD, Smoking, age (overlapping variables filtered by three machine learning methods); Model 3: Adjusted for CVD, Smoking, age, ALT, Heart disease, Lymphocyte, Neutrophil, Total cholesterol, AST, Monocyte, ALP, Platelet, Marital status, Hypertension, Diabetes mellitus, PIR (all variables filtered by three machine learning methods). The Wenzhou cohort model is adjusted to: Model 1: No adjustments were made; Model 2: Adjusted for HPCI (overlapping variables filtered by three machine learning methods); Model 3: Adjusted for HPCI, Education, Eosinophil granulocyte, ALP (all variables filtered by three machine learning methods).

### Abbreviations: COPD, chronic obstructive pulmonary disease; PPV, positive predictive value; NPV, negative predictive value; PIR, poverty-to-income ratio; HPCI, household per capita annual income; FLI,fatty liver index; BODE, body mass index, airflow obstruction, dyspnea, exercise capacity index.

**Supplementary Table 4.** Sensitivity analysis for the association between FLI and COPD using commonly recognized confounding factors

| Variables | OR (95% CI) | *p*-value |
| --- | --- | --- |
| **Wenzhou 2018.2.1-2022.7.5** | |  |
| BODE ≥ 5 | 0.522 (0.300-0.908) | **0.021** |
| **NHANES 2017-2020** | |  |
| COPD | 1.010 (1.003-1.018) | **0.009** |

Abbreviations: COPD, chronic obstructive pulmonary disease; BODE, body mass index, airflow obstruction, dyspnoea, exercise capacity index; PIR, poverty-to-income ratio; GGT, gamma-glutamyl transferase.

In NHANES, we adjusted for age, sex, race/ethnicity, smoking status, socioeconomic status (education and PIR), and major cardiometabolic comorbidities (hypertension, diabetes, and cardiovascular disease). In the Wenzhou cohort, we adjusted for age, sex, smoking status, and major comorbidities (hypertension, diabetes, and heart disease). We did not adjust for the components used to compute FLI (BMI, waist circumference, triglycerides, and GGT) to avoid overadjustment and collinearity.

**Supplementary Table 5.** Sensitivity analysis after overlap weighting in the NHANES cohort

| Analysis | OR (95% CI) | *p*-value |
| --- | --- | --- |
| Univariable logistic regression | 1.009 (1.005-1.013) | **<0.001** |
| Multivariable logistic regression | 1.012 (1.008-1.017) | **<0.001** |

We adjusted for CVD, Smoking, age, ALT, Heart disease, Lymphocyte, Neutrophil, Total cholesterol, AST, Monocyte, ALP, Platelet, Marital status, Hypertension, Diabetes mellitus, PIR.

**Supplementary Table 6.** Sensitivity analysis of FLI categories for COPD prevalence in the NHANES cohort

| Variables | Model 1 | |  | Model 2 | |  | Model 3 | |
| --- | --- | --- | --- | --- | --- | --- | --- | --- |
|  | OR (95% CI) | *p*-value |  | OR (95% CI) | *p*-value |  | OR (95% CI) | *p*-value |
| **NHANES 2017-2020** | | | | | | | | |
| COPD | | | | | | | | |
| Categories |  |  |  |  |  |  |  |  |
| Q1 | Ref | |  | Ref | |  | Ref | |
| Q2 | 1.585 (0.938-2.678) | 0.085 |  | 1.715 (0.980-3.001) | 0.059 |  | 1.529 (0.837-2.793) | 0.167 |
| Q3 | 2.084 (1.206-3.601) | **0.009** |  | 2.755 (1.513-5.017) | **< 0.001** |  | 2.404 (1.265-4.567) | **0.007** |

Abbreviations: COPD, chronic obstructive pulmonary disease; BODE, body mass index, airflow obstruction, dyspnea, exercise capacity index.

FLI categories were defined according to Bedogni et al. : Q1: FLI < 30; Q2: FLI 30–59; Q3: FLI ≥ 60. The NHANES cohort model is adjustd to: Model 1: No adjustments were made; Model 2: Adjusted for CVD, smoking, age (overlapping variables filtered by three machine learning methods); Model 3: Adjusted for CVD, Smoking, age, ALT, Heart disease, Lymphocyte, Neutrophil, Total cholesterol, AST, Monocyte, ALP, Platelet, Marital status, Hypertension, Diabetes mellitus, PIR (all variables filtered by three machine learning methods).

**Supplementary Table 7**. Bootstrap validation of the threshold effect between FLI and COPD severity in the Wenzhou Cohort

| Parameter | Value | 95% Confidence Interval |
| --- | --- | --- |
| **Threshold analysis** |  |  |
| Estimated inflection point | 0.381 | 0.042-1.833 |
| Bootstrap median threshold | 0.381 | - |
| Valid bootstrap samples | 1000/1000 | - |
| **Model comparison** |  |  |
| Log-likelihood ratio | 7.417 | - |
| Log-likelihood ratio *p*-value | **0.025** | - |

Abbreviations: COPD, chronic obstructive pulmonary disease; FLI, fatty liver index.

**Supplementary Figures**

### Supplementary Figure 1. Multiple interpolation of missing data in the Wenzhou cohort and heatmap of the correlations among covariates


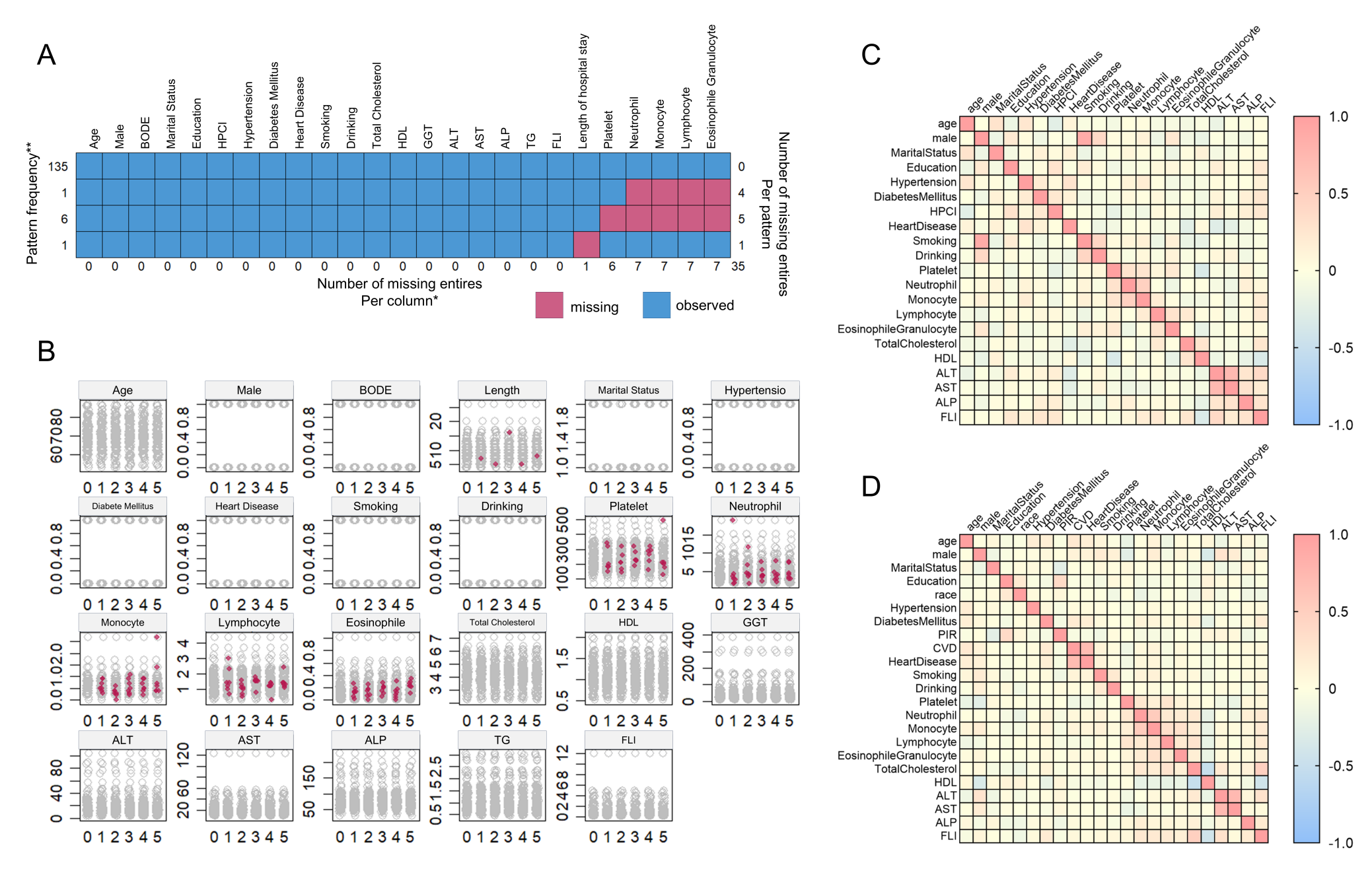


Note: (A) Missing data pattern plot. (B) In the plot of the imputed data, grey denotes observed data, and red signifies imputed data. (C) A heatmap of the correlations among covariates in the Wenzhou cohort. (D) A heatmap of the correlations among covariates in the NHANES cohort.

**Supplementary Figure 2.** Characteristic selection and correlation analysis of FLI and COPD in the NHANES cohort


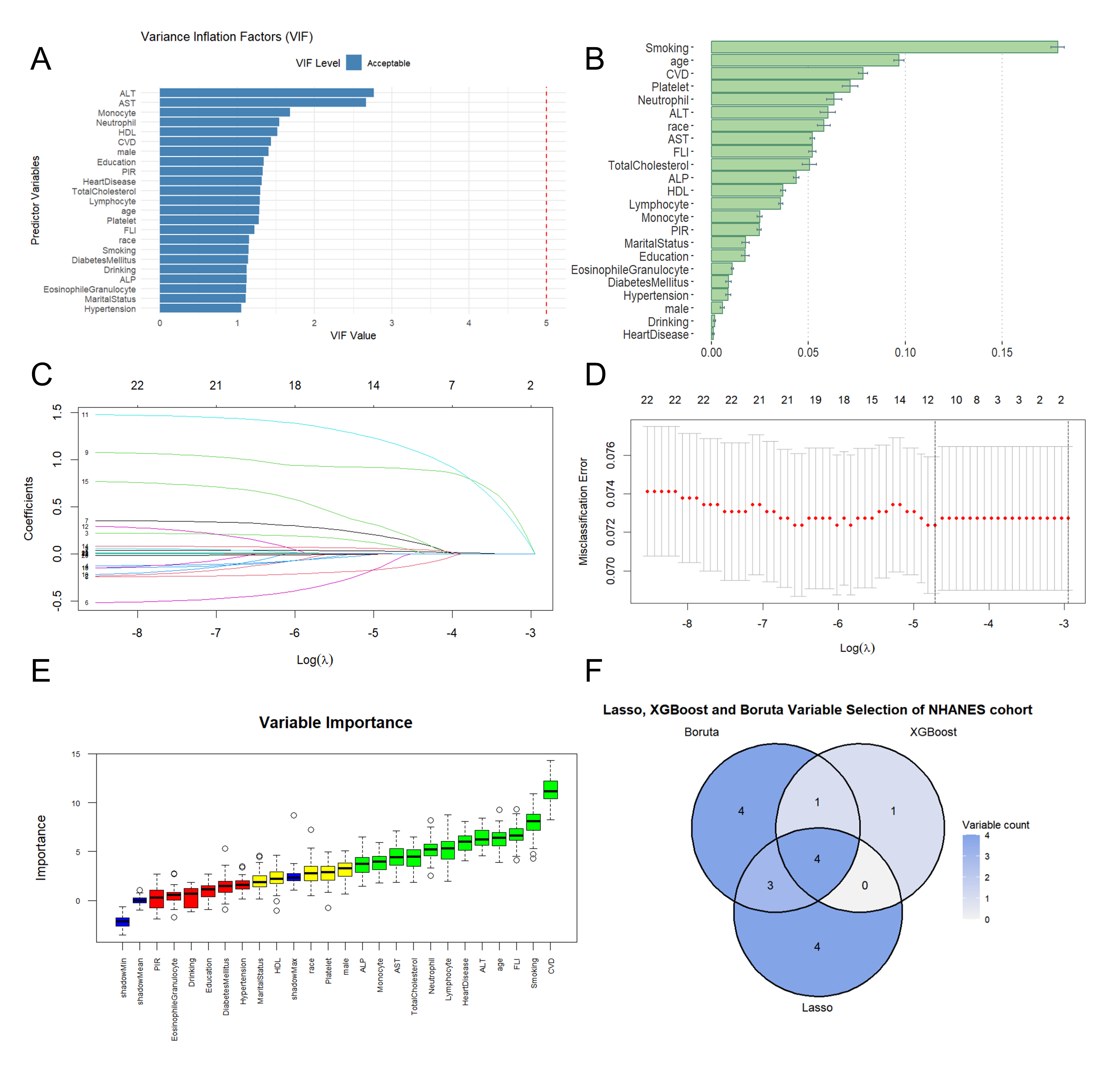


Note: (A) Conduct Variance Inflation Factor (VIF) detection for all variables. (B) Variable importance plot for an XGBoost model. (C) LASSO regression path plot. (D) LASSO 10-fold cross-validation plot. (E) Figure shows the results of feature selection after computerized processing by Boruta algorithm. (F) Three algorithmic Venn diagram screening variables.

**Supplementary Figure 3**. Nomogram for the diagnosis of COPD


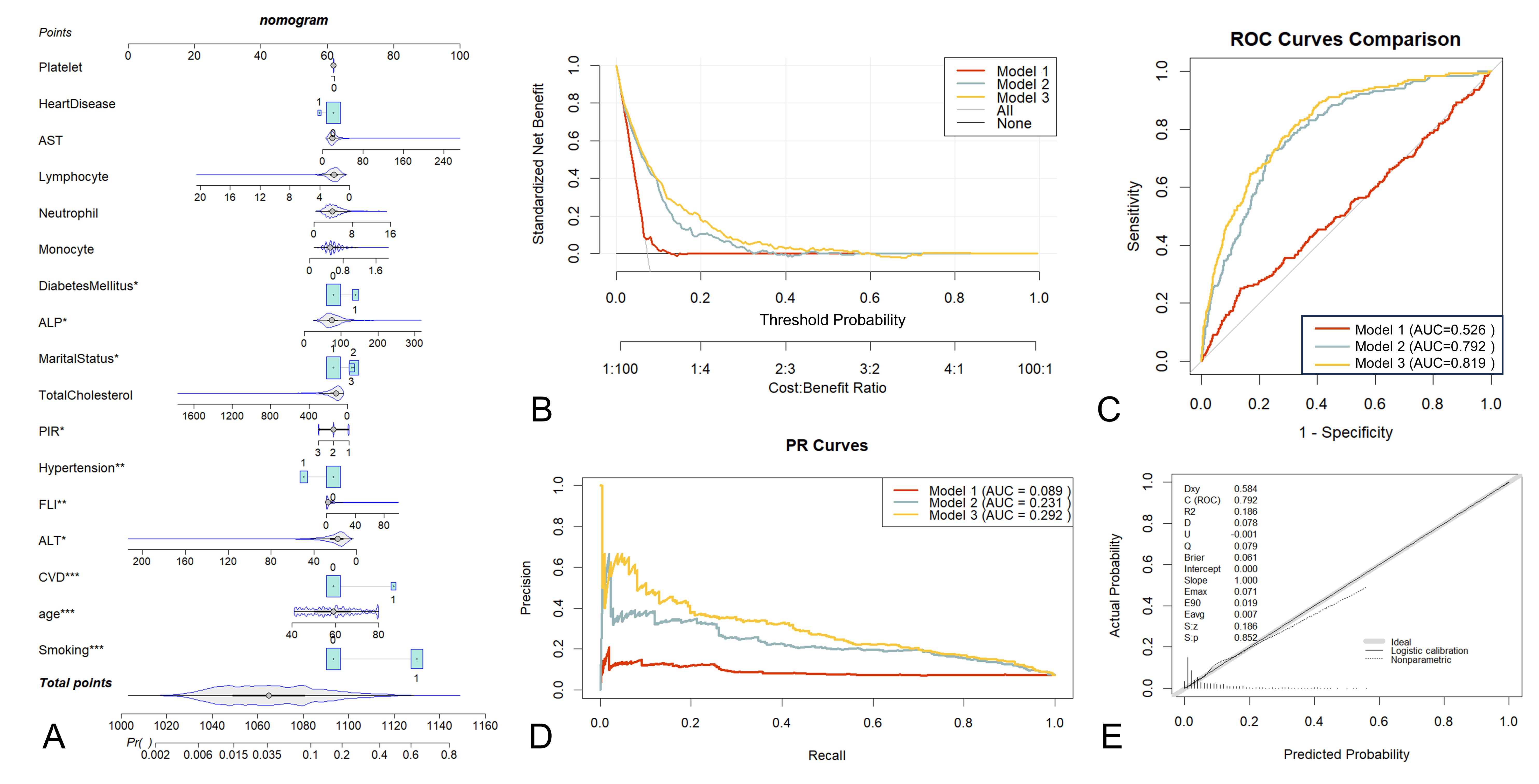


The NHANES cohort model is adjustd to: Model 1: No adjustments were made; Model 2:Adjusted for CVD, smoking, age (overlapping variables filtered by three machine learning methods); Model 3: Adjusted for CVD, smoking, age, ALT, heart disease, lymphocyte, neutrophil, total cholesterol, AST, monocyte, ALP, platelet, marital status, hypertension, diabetes mellitus, PIR (all variables filtered by three machine learning methods).

Note: (A) Nomogram for the diagnosis of COPD. (B) Decision curve. (C) ROC curve (D) PR curve for diagnosis of COPD. (E) Calibration curve for prediction accuracy.

**Supplementary Figure 4.** Comparison of standardized mean differences (SMD) of covariates before and after overlap weighting in the NHANES cohort

**
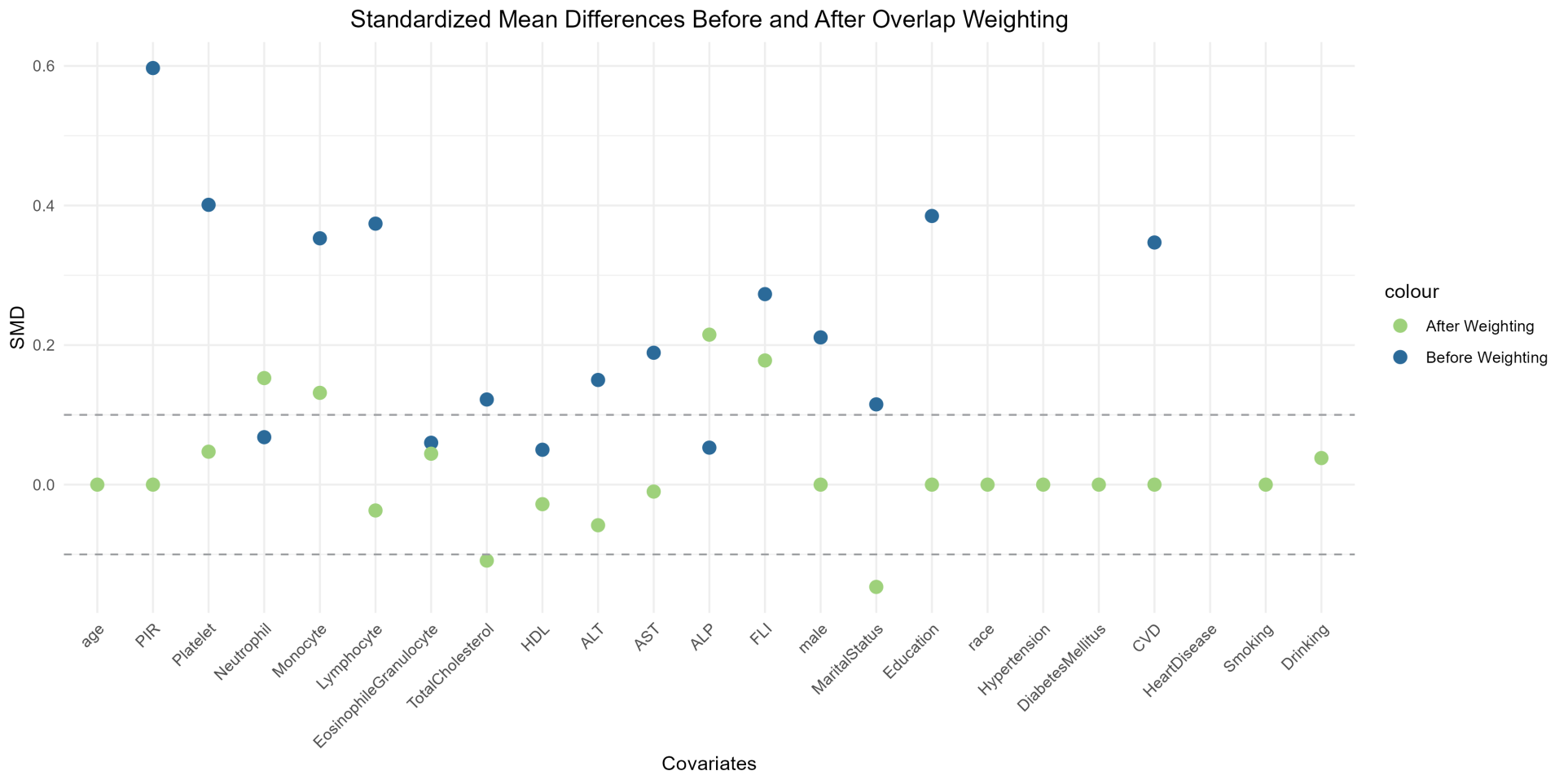
**

Note: The horizontal dashed gray lines indicate the SMD threshold of ±0.2, a commonly used criterion for judging covariate balance (SMD < 0.2 suggests acceptable balance). Blue dots represent SMD values before weighting, and green dots represent SMD values after weighting. After applying overlap weighting, the SMD of most covariates was reduced to below 0.2, indicating that the distribution of baseline covariates between the two groups was effectively balanced.

Abbreviations: COPD, chronic obstructive pulmonary disease; HDL-C, high-density lipoprotein cholesterol; ALT, alanine aminotransferase; ALP, alkaline phosphatase; AST, aspartate aminotransferase; GGT, gamma-glutamyl transferase; BMI, body mass index; FLI, fatty liver index; HPCI, household per capita annual income; BODE, body mass index, airflow obstruction, dyspnoea, exercise capacity index; PIR, poverty-to-income ratio; CVD, cardiovascular disease.

**Supplementary Figure 5**. Internal validation of model performance using K-Fold Cross-Validation in the NHANES and Wenzhou cohorts


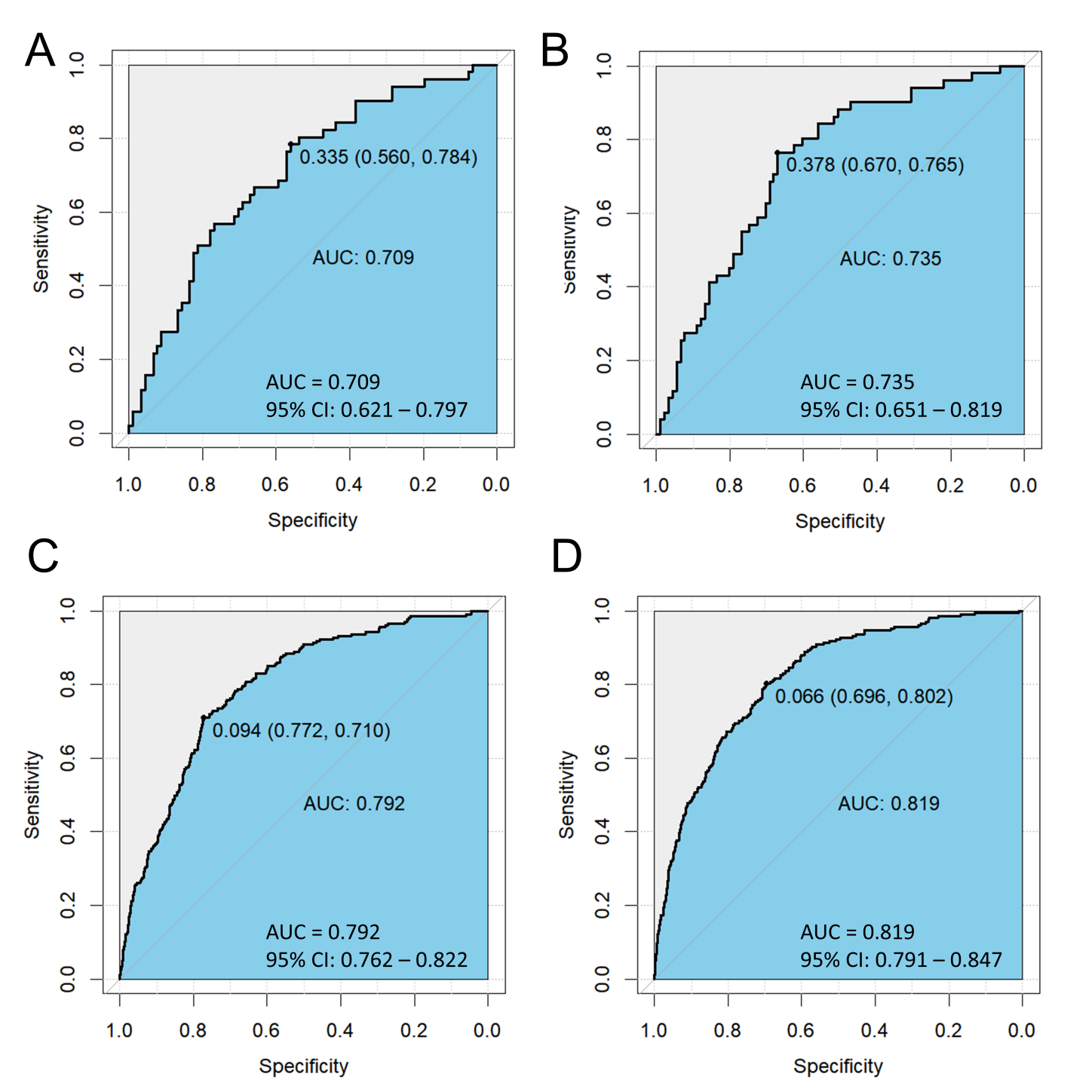


Note: The receiver operating characteristic (ROC) curves illustrate the cross-validated performance of the multivariable logistic regression models. Ten-fold cross-validation was performed in the NHANES cohort, whereas five-fold cross-validation was employed in the Wenzhou cohort due to its smaller sample size. The area under the curve (AUC) values with 95% confidence intervals (CIs) are presented for Model 2 (Panel A) and Model 3 (Panel B) in the Wenzhou cohort, and for Model 2 (Panel C) and Model 3 (Panel D) in the NHANES cohort. These findings confirm the stability and internal validity of the models.
